# Methodological Tutorial Series for Epidemiological Studies: When and How to Split the Follow-up Time in the Analysis of Epidemiological or Clinical Studies With Follow-ups

**DOI:** 10.2188/jea.JE20240245

**Published:** 2025-04-05

**Authors:** Masao Iwagami, Miho Ishimaru, Yoshinori Takeuchi, Tomohiro Shinozaki

**Affiliations:** 1 Department of Digital Health, Institute of Medicine, University of Tsukuba, Ibaraki, Japan; 2 International Institute for Integrative Sleep Medicine (IIIS), University of Tsukuba, Ibaraki, Japan; 3 Pharmaceuticals and Medical Devices Agency, Tokyo, Japan; 4 Faculty of Epidemiology and Population Health, London School of Hygiene and Tropical Medicine, London, UK; 5 Department of Dental Public Health, Graduate School of Medical and Dental Sciences, Institute of Science Tokyo, Tokyo, Japan; 6 Department of Data Science, School of Data Science, Association of International Arts and Sciences, Yokohama City University, Kanagawa, Japan; 7 Department of Information and Computer Technology, Faculty of Engineering, Tokyo University of Science, Tokyo, Japan

**Keywords:** cohort study, self-controlled case series, hazard ratio, time-varying exposure, time-varying confounder

## Abstract

In epidemiological or clinical studies with follow-ups, data tables generated and processed for statistical analysis are often of the “wide-format” type, consisting of one row per individual. However, depending on the situation and purpose of the study, they may need to be transformed into the “long-format” type, which allows for multiple rows per individual. This tutorial clarifies the typical situations wherein researchers are recommended to split follow-up times to generate long-format data tables. In such applications, the major analytical aims consist of (i) estimating the outcome incidence rates or their ratios between ≥2 groups, according to specific follow-up time periods; (ii) examining the interaction between the exposure status and follow-up time to assess the proportional hazards assumption in Cox models; (iii) dealing with time-varying exposures for descriptive or predictive purposes; (iv) estimating the causal effects of time-varying exposures while adjusting for time-varying confounders that may be affected by past exposures; and (v) comparing different time periods within the same individual in self-controlled case-series analyses. This tutorial also discusses how to split follow-up times according to their purposes in practical settings, providing example codes in Stata, R, and SAS.

## INTRODUCTION

In epidemiological studies or clinical studies with follow-ups, that leverage either primary data collection (as observational or interventional studies) or the secondary use of routinely collected electronic health records, the data tables generated and processed for statistical analysis are often of the “wide-format” type, consisting of one row per individual. Such tables are used for performing many statistical analyses. However, depending on the specific research question and data availability, researchers may benefit from transforming their data tables into the “long-format” type—which allows for multiple rows per individual—by splitting the follow-up time. As this tutorial will clarify, these long-format data tables can facilitate flexible survival analyses via standard statistical software.

This tutorial aims to clarify when and how to split the follow-up times in epidemiological studies or clinical studies with follow-ups, according to the five major analytical aims, in distinct applications (Box 1). To explain each situation and its most appropriate analytical procedure, we used publicly available example datasets (“Gehan survival data,” provided by Gehan et al,^1^ “BMT” dataset provided by Zabor,^2^ and “MMR and meningitis in Oxford” example provided by Farrington et al^3^) with the original column names and order preserved, in addition to our hypothetical annual health survey dataset concerning the association between time-updated body mass index (BMI) and death. The relevant commands for Stata (version 17; Stata Corp, College Station, TX, USA), R (version 4.4.0; R Foundation for Statistical Computing, Vienna, Austria), and SAS (version 9.4; IBM Corp, Armonk, NY, USA) are provided in eMaterial 1, eMaterial 2, and eMaterial 3. Note that we focus on the analysis of the first event in cohort studies (ie, cohort studies with either primary data collection or secondary use of electronic health records, including registry and claims data). The analysis of multiple events is beyond the scope of this tutorial paper, except for the self-controlled case series (SCCS), where multiple events can be analyzed with the provided commands under the (strong) assumption that the events are independent with each other.

**Box 1.** The five situations with distinct analytical aims discussed in this tutorial, and their corresponding datasets

(i)	Estimating outcome incidence rates and their ratios between two (or more) groups according to specific follow-up time periods (“Gehan survival data”)
(ii)	Assessing the proportional hazards assumption in Cox models (“Gehan survival data”)
(iii)	Dealing with time-varying exposures for descriptive and predictive purposes (“BMT” dataset as an example for binary variables, and our hypothetical annual health survey dataset as an example for continuous ones)
(iv)	Estimating the causal effects of time-varying exposures while adjusting for time-varying confounders that may be affected by past exposures (no example data illustration)
(v)	Comparing different time periods within the same individual in self-controlled case series analyses (“MMR and meningitis in Oxford” dataset)

## SITUATION 1: ESTIMATING OUTCOME INCIDENCE RATES AND THEIR RATIOS ACCORDING TO SPECIFIC FOLLOW-UP TIME PERIODS

### Nonparametric, parametric, and semiparametric perspectives for estimating rates and rate ratios

**Figure 1.**
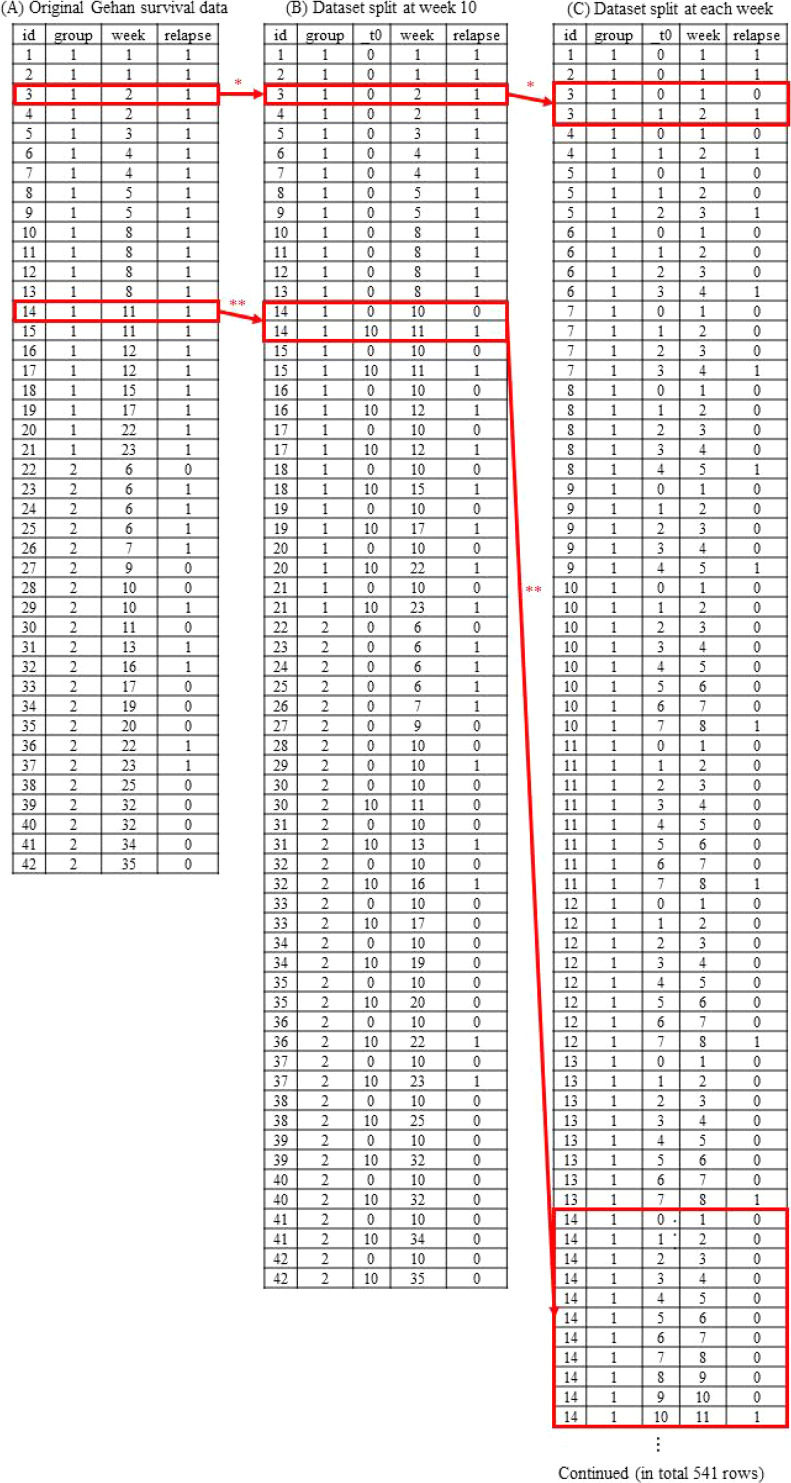
Data tables of original Gehan survival data (**A**), and datasets split at week 10 (**B**), and at each week (**C**). ^*^For the third patient with a follow-up period of 2 weeks, the row is not split in the data table (**B**) because the follow-up length is ≤10 weeks but is split into two rows within the data table (**C**). ^**^For the 14^th^ patient with a follow-up period of 11 weeks, the row is split into two rows before and after 10 weeks in the data table (**B**) and is therefore split into 11 rows in the data table (**C**).

When conducting survival analyses in cohort studies, researchers often estimate outcome incidence rates in ≥2 groups and rate ratio(s) between them, with or without adjustment for potential confounders. To illustrate this case, we can use the publicly available Gehan survival dataset, by Gehan et al, as an example (Figure 1A).^1^ Briefly, the data table consists of 42 rows for 42 patients with leukemia, comprising 21 and 21 patients who did and did not receive mercaptopurine treatment as an exposure of interest (recorded in the “group” column, where 1 indicates unexposed and 2 indicates exposed), who were followed from weeks 0–35 (“week”), of which 30 patients exhibited leukemia relapse as an outcome of interest (recorded in the “relapse” column, where 0 indicates censoring before the occurrence of the outcome and 1 indicates the outcome observed during the follow-up period). Patient identification numbers (ie, the “id” column) were arbitrarily added by us.

**Figure 2.**
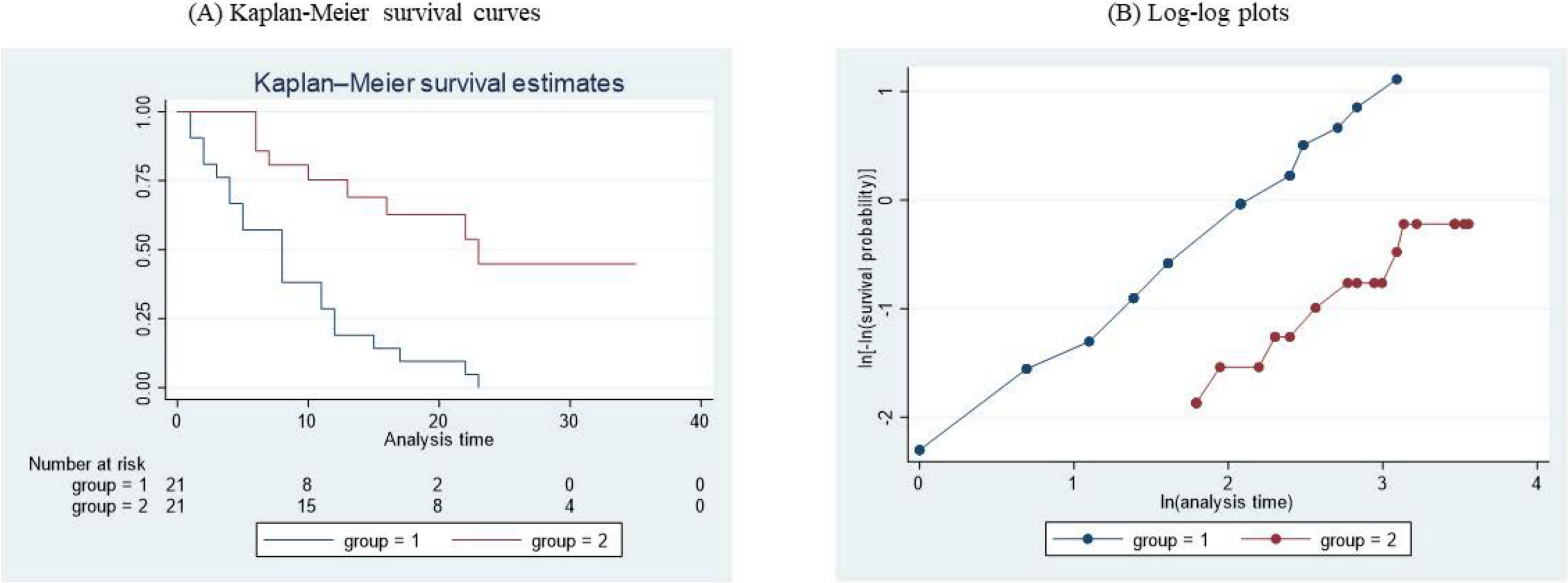
Kaplan–Meier survival curves (**A**) and log-log plots (**B**) of the Gehan survival data. ln = log. Note: The graphs were made by Stata (version 17).

The first approach to summarize the survival outcome data is the nonparametric one. The Kaplan–Meier survival curves are shown in Figure 2A, and a log-rank test yielded a *P* value of <0.001, which suggests that the survival curves differ between the groups within the follow-up period.

Another approach for summarizing this survival outcome data consists of calculating incidence rates using the person-time method. When stratified by exposure status, the incidence rates were 0.115 cases/person-week (or 21 cases/182 person-weeks) and 0.025 cases/person-week (or 9 cases/359 person-weeks) in the unexposed and exposed groups, respectively. Although the incidence rates were calculated nonparametrically, their standard errors and confidence intervals (CIs) can be parametrically estimated through a Poisson distribution for outcome counts given the observed person-time, which is equivalent to assuming an exponential distribution for each time to outcome. For example, the standard error of log incidence rates in the unexposed group is estimated as the square root of 1/21 (or 0.218), and the corresponding 95% CI is given by: exp[log(0.115) ± 1.96(0.218)]—or 0.075–0.177 cases/person-week.

A parametric, unadjusted Poisson regression model, including an exposure variable and log of follow-up time as an “offset” variable, directly provides a rate ratio (exposed vs unexposed) of 0.217 (= 0.025/0.115) with a 95% CI ranging between 0.100 and 0.474, which can be obtained through manual calculation of exp[log(0.217) ± 1.96(0.398)], where 0.398 (or the square-root of [1/9 + 1/21]) represents the estimates of the standard error of the log-rate ratio. Moreover, when required, Poisson regression models can estimate confounder-conditional (ie, adjusted) rates and rate ratios, by including confounders as covariates.

If we want to drop the parametric assumption of constant rates in each of the groups imposed by Poisson distribution, we can use Cox regression models (also known as proportional hazards models) as semiparametric methods. The time-varying instantaneous rates at each time point are referred to as hazards. Although Cox regression models do not assume that hazards are constant or specific functions of time (eg, linearly increasing or decreasing hazards), they require that the ratio of hazards between exposed and unexposed groups remain constant over time (ie, the proportional hazards assumption). The unadjusted Cox model for the same Gehan survival dataset yielded a crude hazard ratio of 0.221 (95% CI, 0.099–0.493). Example commands that can be used to calculate this in Stata, R, and SAS are shown in eMaterial 1, eMaterial 2, and eMaterial 3.

### Splitting follow-up data to estimate period-specific rates and rate ratios

Researchers may be interested in estimating the outcome incidence rates or their ratios according to specific follow-up time periods if, within that interval, (i) the hazard is considered to be approximately constant (when using the Poisson models), (ii) the proportional hazards assumption is considered to approximately hold (when using the Cox models), or (iii) the period-specific rate (ratio) itself is considered to be of clinical interest.

For example, if one wishes to estimate rates and rate ratios before and after 10 weeks in the Gehan survival dataset, the original data table should first be split at 10 weeks. Using the Stata, R, and SAS commands detailed in eMaterial 1, eMaterial 2, and eMaterial 3, any rows for patients with ≥10-week follow-up periods are split into two rows, containing information for before and after 10 weeks, respectively—whereas any rows for patients with <10 weeks of follow-up remain unchanged (Figure 1B). Restricting the survival analysis to rows before or after 10 weeks, the outcome incidence rates and their ratio can then be estimated according to the specific follow-up time period. Before 10 weeks, the estimated incidence rates were 0.094 cases/person-week (or 13 cases/139 person-weeks) and 0.026 cases/person-week (or 5 cases/190 person-weeks) in the unexposed and exposed groups, respectively, with a rate ratio of 0.281 (95% CI, 0.100–0.789) and a hazard ratio of 0.280 (95% CI, 0.100–0.788). After 10 weeks, the corresponding incidence rates are 0.186 cases/person-week (8 cases/43 person-weeks) and 0.024 cases/person-week (4 cases/169 person-weeks), with a rate ratio of 0.127 (95% CI, 0.038–0.422) and a hazard ratio of 0.164 (95% CI, 0.048–0.556).

However, caution is advised when interpreting the latter hazard ratio estimates as causal estimates, because their follow-up starts at a splitting time point (eg, 10 weeks) and they are conditional on not having the outcome or any censoring until this time point.^4^^,^^5^ Accordingly, the latter estimates may suffer from selection bias unless we condition on all risk factors of the outcome or expect a null exposure effect, which is referred to as “built-in survivor bias” in hazard ratios.^4^^,^^5^ Such bias can arise even if the unconditioned risk factors are not confounders at the baseline.

## SITUATION 2: ASSESSING THE PROPORTIONAL HAZARDS ASSUMPTION IN COX MODELS

Cox regression models are based on the proportional hazards assumption, which means that the hazard ratios between ≥2 groups within the strata of covariates are constant over time. As mentioned above, hazard ratios themselves may lack causal interpretation, as they inherently include covariate-unbalanced risk-set selection (ie, hazards in exposed and unexposed groups are typically conditional on different sets of non-diseased subpopulations with distinct susceptibilities to disease).^4^^,^^5^ Moreover, the proportionality of hazards often requires unrealistic causal relationships behind exposures and outcomes, such as the absence of delayed effects or variations in disease susceptibility.^6^

Bearing this in mind, assessing whether the proportional hazards assumption approximately holds may be useful during the practical application of Cox models. Several main methods are used to examine deviations from the proportional hazards assumption.

### Graphical assessment of transformed survival estimates and the Schoenfeld residuals

The first of these approaches is the visualization of log-log survival plots, where log[−log(survival probability at time t)] is plotted against log(t)—that is, the transformed version from the two axes of the Kaplan–Meier plots (Figure 2B). In the Gehan survival dataset, the roughly parallel log-log survival plots for the two groups suggest that there is little evidence against the proportional hazards assumption in the univariable Cox model.

Another approach is to plot the Schoenfeld residuals (ie, the “partial residuals,” according to the original article by Schoenfeld et al^7^) and test, versus the null hypothesis, whether the distributions of the Schoenfeld residuals are constant over time under the proportional hazards assumption. In the current example, a large *P* value of around 0.89 (Note: aligning the default setting, Stata, R, and SAS yield not identical but very similar values) was obtained, suggesting little evidence against the proportional hazards assumption. However, because any *P* value is highly sensitive to the sample size of dataset, the large *P* value itself does not verify (or even support) the proportionality of hazards, particularly in small datasets.^8^

### Assessing interactions with time in split datasets

Another approach is to compare time-specific hazard ratios (such as those obtained in the previous section for the example Gehan survival dataset being analyzed) over the follow-up period. To make this type of comparison, one can examine whether the hazard ratios remain constant over time by assessing the interaction term between exposure and the indicator of the time interval in a single Cox model stratified by the time interval.

First, the follow-up period may be arbitrarily or reasonably (eg, in reference to log-log survival plots) split into ≥2 intervals. To illustrate this in the Gehan survival dataset, using the split data table shown in Figure 1B, a single Cox model was fit by including the split follow-up time indicator (ie, the binary variable indicating <10 or ≥10 weeks) as a stratified variable, and the exposure status and their interaction term as covariates. This is equivalent to comparing the hazard ratios before versus after 10 weeks that would be estimated using Cox models separately fitted in datasets with <10 and ≥10 weeks. The coefficient of the interaction term corresponds to the ratio of hazard ratios, which was estimated to be 0.584 (95% CI, 0.118–2.902, *P* = 0.511), suggesting that the hazard ratios before and after 10 weeks were not substantially different.

The interaction between the exposure status and follow-up time can be modeled continuously by splitting the follow-up time at all possible time points (ie, at any week between 0 and 35 in the Gehan survival dataset—but only those time points at which the outcome occurs are actually used for analysis—as is illustrated in Figure 1C) and treating the split time points as a continuous variable in the Cox model. The interaction term between exposure and the linear term of the time points then assesses the linear trend in hazard ratios over time. The Gehan survival data yielded a change (ratio) in hazard ratios of 1.008 (95% CI: 0.894–1.137, *P* = 0.894) per week, which suggests little evidence that the hazard ratio is linearly increasing or decreasing over time. However, this *P* value only measures the compatibility of linear changes in hazard ratios over the follow-up period.

To assess the proportional hazards assumption in the Cox models, researchers should not rely on any single *P* value for “testing” the proportional hazards assumption, because such an assumption does not strictly hold in any dataset. Rather, one should carefully check the log-log survival or the Schoenfeld residual plots, and the estimates of “interactions” with time, to ensure that there are no severe deviations from the assumed models.^8^

## SITUATION 3: DEALING WITH TIME-VARYING EXPOSURES FOR DESCRIPTIVE AND PREDICTIVE PURPOSES

### Summarizing and comparing outcome incidence rates according to time-varying exposure status

**Figure 3.**
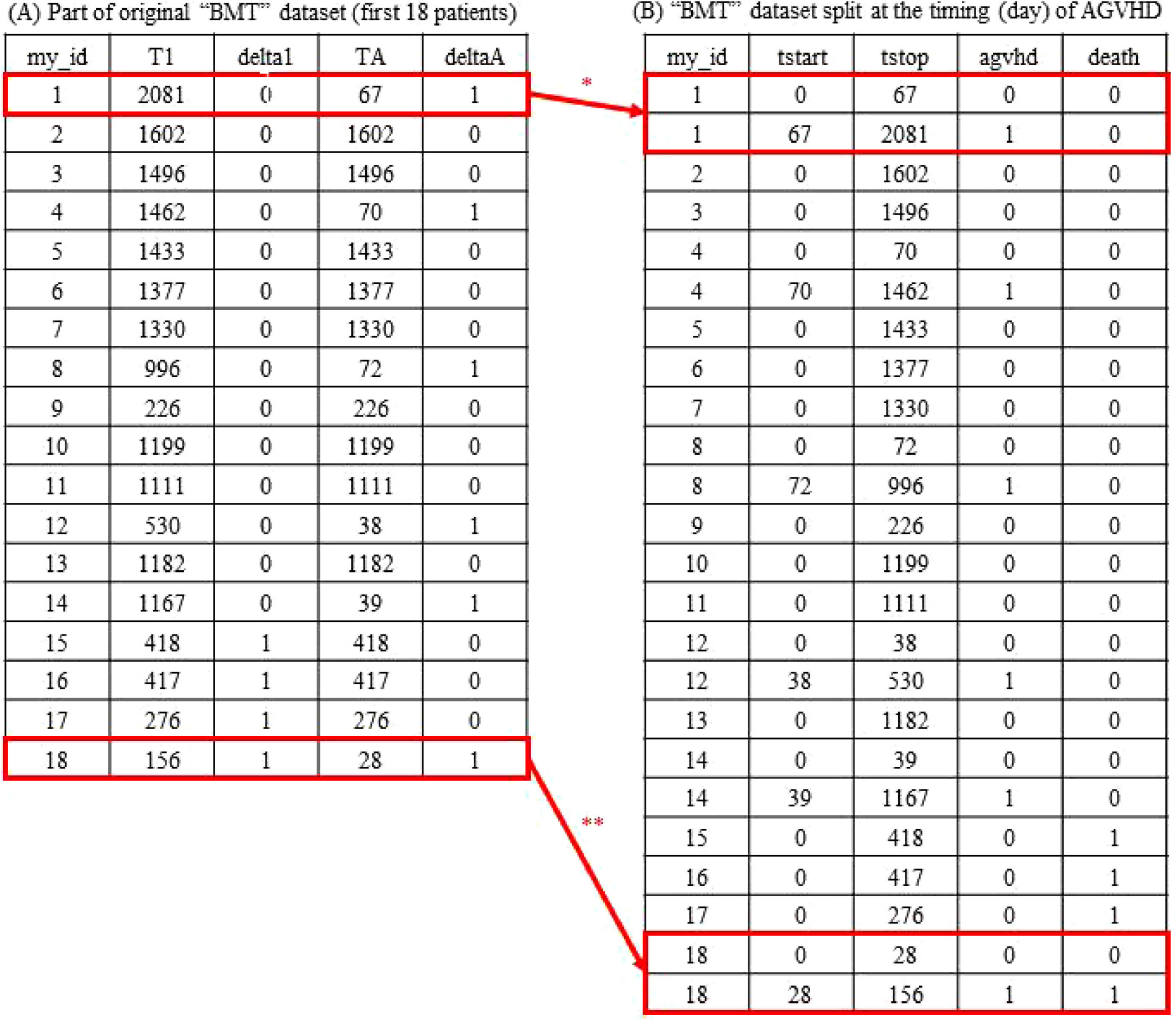
Data tables of a subset (the first 18 patients) of the original “BMT” dataset (**A**), and the dataset split according to the timing of acute graft-versus-host disease (AGVHD) onset (**B**). ^*^For the first patient, the row is split into two rows: before and after AGVHD onset. ^**^For the 18^th^ patient, the row is split into two rows—before and after the onset of AGVHD—where the death status is 0 in the first row and 1 in the second. AGVHD, acute graft-versus-host disease.

In cohort studies, exposures may occur (the exposure status may change) after the start of the follow-up. For example, the presence or absence of acute graft-versus-host disease (AGVHD) following bone marrow transplantation (BMT) in patients with leukemia can serve as a time-varying exposure, whereas death in such cases represents an outcome of interest.^2^ Figure 3A describes a portion of the publicly available “BMT” dataset by Zabor.^2^ This subset consists of the first 18 of 137 patients with leukemia who received BMT and includes variables such as patient ID (“my_id”), the length (days) of follow-up (“T1”), the presence or absence of death (“delta1”) and AGVHD (“deltaA”) during the follow-up period, and the timing (day) of AGVHD (“TA,” which is equal to “T1” for patients without AGVHD).

The first step when analyzing this type of dataset is to summarize the incidence rates of outcomes (eg, death) according to the exposure status. To estimate the incidence rates of death by time-updated AGVHD status, the data table should be transformed into Figure 3B by splitting a row for a participant with AGVHD into two rows—before and after the occurrence of AGVHD—using the commands shown in eMaterial 1, eMaterial 2, and eMaterial 3. The incidence rates before and after the occurrence of AGVHD can then be directly calculated by filtering the rows with “agvhd” equal to 0 (before AGVHD) and the rows with “agvhd” equal to 1 (after AGVHD). These incidence rate ratios may then be compared using the unadjusted (univariable) Poisson regression model with “agvhd” as a predictor variable and the log of the follow-up time (ie, the difference between “tstop” and “tstart” in Figure 3B) as an offset variable. If we wish to drop the parametric assumption of constant hazards of death before and after the occurrence of AGVHD, we can use the unadjusted time-dependent Cox model in a “counting process” format, specifying the start (“tstart”) and end (“tstop”) of each exposure period, to estimate hazard ratios between periods with and without AGVHD.

### Estimating short-term and long-term associations between time-varying exposures and the outcome

**Figure 4.**
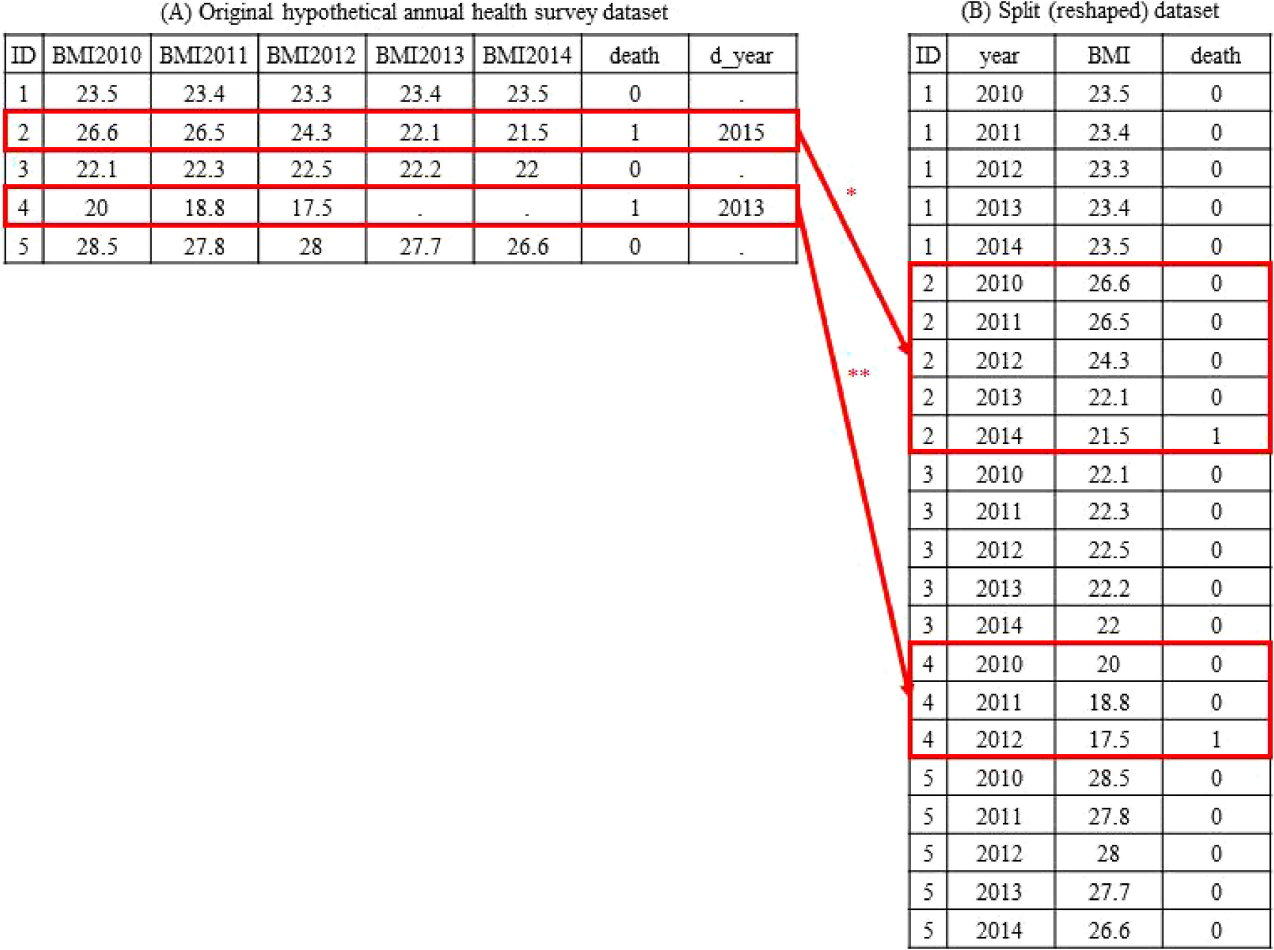
Data tables for the original hypothetical annual health survey dataset (**A**) and the split (reshaped) dataset (**B**). ^*^For the second patient, the row is split into five rows for each survey year between 2010 and 2014, with death in the last row because the patient died during the 2015 survey. ^**^For the fourth patient, the row is split into three rows in each survey year between 2010 and 2012, with death in the last row because the patient was dead at the time of the 2013 survey.

In cohort studies, repeated measurements of variables may be taken during follow-up periods, wherein exposure information can be updated from baseline values. As an example, Figure 4A shows a hypothetical annual health survey dataset with five rows for five individuals (indexed by “ID”), including BMIs measured annually between 2010 and 2014 (“BMI2010” through “BMI2014”). Death status (“death,” with 1 indicating dead and 0 indicating alive) and its associated year (“d_year”) are recorded.

If researchers are interested in the association across the entire length of follow-up (called “long-term” association^9^^,^^10^) between BMI at the first survey year (2010) and death, they can apply the discrete-time Cox model to estimate the hazard ratio of “BMI2010” for death over the entire follow-up period.

Meanwhile, if researchers are interested in the “short-term” (defined as the interval between successive updates of exposures^9^^,^^10^) association between the time-updated BMI and death, the discrete-time Cox model (which is equivalent to the pooled logistic model) including “BMI” as a covariate can be fitted. The transformed long-format data table shown in Figure 4B can be used to estimate a weighted average of time window-specific hazard ratios of BMI at each year on death during the following year.^9^^,^^10^

### Estimating multivariable-adjusted associations for descriptive and predictive purposes

Researchers may want to describe the multivariable-adjusted association between an exposure and an outcome or develop a prediction model for the outcome based on the multiple variables (especially using biomarkers). For these purposes, time-varying exposures and covariates can be straightforwardly incorporated into the multivariable models using long-format data such as Figure 3B or Figure 4B.

Alternatively, researchers may wish to examine the causal relationship between an exposure and an outcome. For example, in the association between BMI and death, potential confounders could include age, sex, the presence of diabetes, and exercise habits. If researchers estimate the long-term association between BMI measured at the time of entry into the cohort and death, adjusting for these confounders measured at or before the time of cohort entry, a hazard ratio for BMI in the multivariable Cox model may be causally interpretable.

However, if researchers wish to estimate the short-term association between time-updated BMI and death, a Cox model adjusting for these confounders measured at or before the time of entry into the cohort is not appropriate, because the time-varying BMI is influenced by time-varying confounders (eg, diabetes, exercise habits) measured during the follow-up period. Meanwhile, the time-dependent Cox model adjusting for time-varying confounders is known to result in biased estimates, mainly caused by “exposure-confounder feedback.”^11^ Another approach, such as the use of g-methods, is necessary in such cases (as is explained in further detail in Situation 4).

## SITUATION 4: ESTIMATING THE CAUSAL EFFECTS OF TIME-VARYING EXPOSURES ADJUSTING FOR TIME-VARYING CONFOUNDERS THAT MAY BE AFFECTED BY PAST EXPOSURES

Researchers may want to evaluate the causal effects of time-varying exposures by adjusting for time-varying confounders that may be affected by past exposures. Illustrative examples of this practice include:

• In a study on zidovudine treatment (ie, a time-varying exposure) on mortality among patients with human immunodeficiency virus, CD4 lymphocyte count was considered as a time-varying confounder that was affected by past exposures, because it is both an independent predictor of zidovudine treatment and survival while being influenced by any prior zidovudine treatments.^12^
• In a study on methotrexate treatment (ie, a time-varying exposure) on mortality in patients with rheumatoid arthritis, the disability index (a measure of a patient’s level of functional ability) was considered to be a time-varying confounder affected by past exposure because it predicts whether a patient with rheumatoid arthritis will be treated with methotrexate and whether they will survive and also because methotrexate treatment lowers the disability index.^13^

To evaluate the causal effects of time-varying exposures in the presence of exposure-confounder feedback, researchers can apply g-methods, such as inverse probability weighting for marginal structural models,^12^ g-estimation of a structural nested model,^14^ and the parametric g-formula.^15^ The full details behind these g-methods are beyond the scope of this tutorial and interested readers are recommended to read other relevant papers^16^^–^^18^ and textbooks on the subject.^19^^–^^21^

To apply g-methods, we recommend that researchers transform their original wide-format data tables into long-format ones consisting of multiple rows at all possible time points per individual, as illustrated in Figure 1C. Depending on the research questions and data availability, the units of time could be days, weeks, 2-week intervals, months, 3-month intervals, years, or any other temporal divisions. With this data structure, researchers can estimate effect measures, such as hazard ratios (which are, by nature, not suitable indicators of causal inference^4^), risk ratios, risk differences, hazard differences, and differences in restricted mean survival times between groups.

## SITUATION 5: COMPARING DIFFERENT TIME PERIODS WITHIN THE SAME INDIVIDUAL IN SELF-CONTROLLED CASE SERIES ANALYSES

Finally, the SCCS is a unique method that requires splitting follow-up times for analysis. The SCCS design was proposed by Farrington et al in 1995 to examine the suspected risk of the Measles-Mumps-Rubella (MMR) vaccine on the incidence of aseptic meningitis.^22^ Since then, it has been used primarily to examine the causal relationship between vaccination and acute outcomes,^23^^,^^24^ although a few researchers have attempted to apply this method to other types of exposures, such as infectious episodes,^24^ drugs,^25^ and surgical procedures.^26^^,^^27^

Full details concerning the SCCS process have been explained elsewhere.^28^^–^^30^ Briefly, only individuals with the outcome of interest are identified and included in the SCCS analysis, as any information concerning those without the outcome is not used for analysis. The study periods for the cases are decided by the researchers, considering their research questions and data availability. A few examples of such custom study periods include between the 1^st^ and 2^nd^ birthdays of the study participants,^31^ and from the date when mass vaccinations for coronavirus disease 2019 began to the most recent date in the available dataset.^32^ Then, individual study periods are split into “risk periods” (during which the risk of outcome is assumed to be increased because of the exposure) and “baseline periods” (comprising the remaining study periods). Individuals can have ≥2 risk periods if they are exposed several times over the study period, whereas they can have ≥2 episodes of the studied outcome. Finally, the incidence of the outcome(s) during the risk and baseline periods are compared in a conditional Poisson regression model to estimate an incidence rate ratio. Although time-independent confounders (eg, sex and genetics) are automatically canceled out by the within-person comparison used in SCCS, time-dependent confounders (eg, age and season) must be explicitly adjusted for in the model.

**Figure 5.**
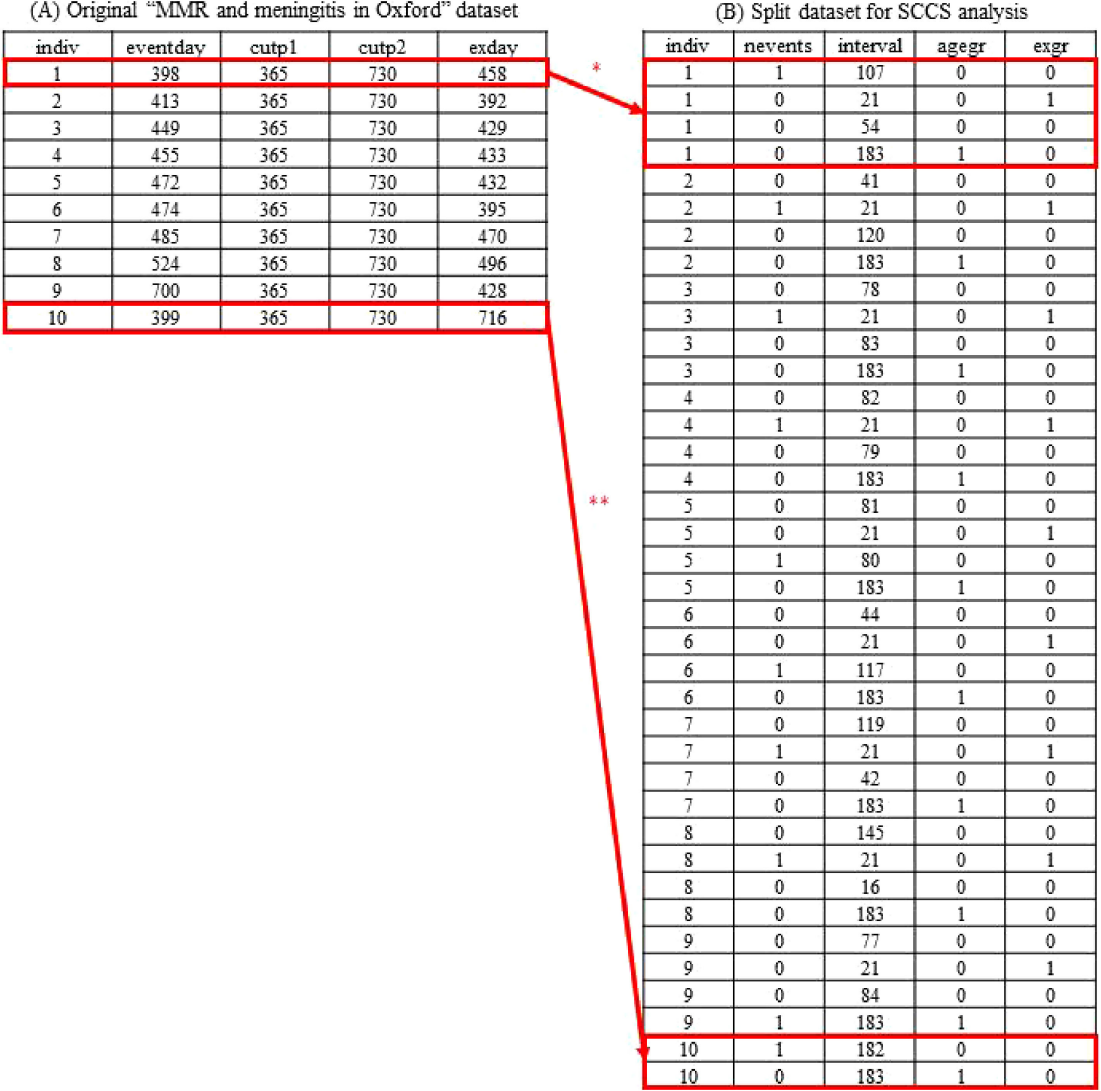
Data tables for the original “MMR and meningitis in Oxford” dataset (**A**) and the dataset split for self-controlled case series analysis (**B**). ^*^For the first patient, the row is split into 4 rows at the 14 and 35-day marks following the patient’s vaccination, to define the “risk period,” and the date when the participant reached the age of 1.5 years, as is illustrated graphically in Figure 6. ^*^For the 10^th^ patient, the row is split into only two rows, at the date when the participant reached the age of 1.5 years because the 14 (ie, day 730) and 35 (ie, day 751) day marks following the patient’s vaccination date (ie, day 716) extended beyond the end of the study period (ie, day 730).

**Figure 6.**
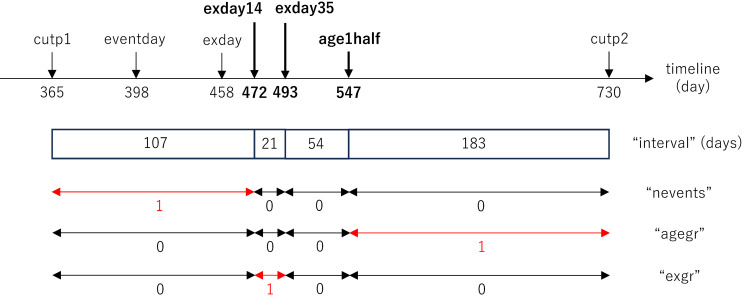
Graphical representation of splitting the time for the first patient in the “MMR and meningitis in Oxford” dataset. Note: For this first patient in the “MMR and meningitis in Oxford” dataset, the row is split into 4 rows at the 14 and 35-day marks following the patient’s vaccination, to define the “risk period,” and the date when the participant reached the age of 1.5 years. The variable names correspond to Figure 5 and are explained in the main draft.

In SCCS, a wide-format dataset consisting of one row per individual should be transformed into a long-format dataset consisting of multiple rows per individual. Figure 5A shows the “MMR and meningitis in Oxford” example dataset provided by Farrington et al,^3^ including 10 rows of 10 children (as suggested in the column named “indiv”), the timing (day) of meningitis onset (“eventday”), and the timing (day) of MMR vaccination (“exday”). Study periods are suggested to range between the 1^st^ (“cutp1,” where all individuals have values of 365) and 2^nd^ birthdays (“cutp2” where all individuals have values of 730) of the study participants. Using the commands shown in eMaterial 1, eMaterial 2, and eMaterial 3, the original data table can be transformed into the one shown in Figure 5B. As is suggested in Figure 6, using the first patient as an example, the individual’s follow-up period was split at 14 days (“exday14”—which is the sum of “exday” + 14) and 35 days after the vaccination date (“exday35”—which is the sum of “exday” + 35) to obtain the risk period, and the date when each participant reached the age of 1.5 years (“age1half,” where has all individuals have values of 547). Finally, the length of each period was calculated and the period during which the outcome occurred was identified. Our STATA, R, and SAS commands (detailed in eMaterial 1, eMaterial 2, and eMaterial 3) differ somewhat from those provided by Farrington et al on their website,^3^ indicating that it is possible to obtain the same data table for this SCCS analysis using different approaches.

### Conclusions

We summarized the typical situations wherein researchers are recommended to split follow-up times during analyses of cohort studies, and how to split the data in different situations. With the current availability of large-scale electronic health records and biobank cohorts with repeated measurements, we believe that epidemiologists and biostatisticians will see increasing opportunities to use these skills to split the follow-up times in their analyses. However, as is indicated in this tutorial, it is key for researchers to consider whether these methods are necessary and which of them is most suitable for answering their research questions.

## SUPPLEMENTARY MATERIAL

eMaterial 1. Commands for Stata (version 17)

eMaterial 2. Commands for R (version 4.4.0)

eMaterial 3. Commands for SAS (version 9.4)
