# Supplementary material for: Methodological Tutorial Series for Epidemiological Studies: When and How to Split the Follow-up Time in the Analysis of Epidemiological or Clinical Studies With Follow-ups
Source: J Epidemiol. 2025 Apr 5;35(4):161–9. doi: 10.2188/jea.JE20240245 (PMC11882348; doi:10.2188/jea.JE20240245)
Supplement: Supplementary file 1 [file je-35-161-s001.pdf]

eMaterial 1. Commands for Stata (version 17)

***Situation 1: estimating outcome incidence rates and their ratios according to specific follow-up time periods**

```
*to input the "Gehan survival data"
clear
input id group week relapse
1 1 1 1
2 1 1 1
3 1 2 1
4 1 2 1
5 1 3 1
6 1 4 1
7 1 4 1
8 1 5 1
9 1 5 1
10 1 8 1
11 1 8 1
12 1 8 1
13 1 8 1
14 1 11 1
15 1 11 1
16 1 12 1
17 1 12 1
18 1 15 1
19 1 17 1
20 1 22 1
21 1 23 1
22 2 6 0
23 2 6 1
24 2 6 1
25 2 6 1
26 2 7 1
27 2 9 0
28 2 10 0
29 2 10 1
30 2 11 0
31 2 13 1
```

```
32 2 16 1
33 2 17 0
34 2 19 0
35 2 20 0
36 2 22 1
37 2 23 1
38 2 25 0
39 2 32 0
40 2 32 0
41 2 34 0
42 2 35 0
end
*to “stset” for survival analysis in Stata
stset week, failure(relapse) id(id)
*to estimate the crude incidence rate overall
strate
*to estimate the crude incidence rate by exposure status
strate group
*to illustrate Kaplan Meyer survival curves
sts graph, by(group) risktable
*to conduct a log-rank test
sts test group
*to estimate a crude rate ratio in an unadjusted Poisson regression analysis
streg group, dist(exp)
*to estimate a crude hazard ratio in an unadjusted Cox regression analysis
stcox group
*to split the data table at 10 weeks
stsplit time, at(0,10)
*to repeat the aforementioned analyses before 10 weeks
strate group if time==0
streg group if time==0, dist(exp)
stcox group if time==0
*to repeat the aforementioned analyses after 10 weeks
strate group if time==10
streg group if time==10, dist(exp)
stcox group if time==10
```

***Situation 2: assessing the proportional hazards assumption in Cox models**

*to newly input the "Gehan survival data" (because the aforementioned data are already split)

```
clear
input id group week relapse
1 1 1 1
2 1 1 1
3 1 2 1
4 1 2 1
5 1 3 1
6 1 4 1
7 1 4 1
8 1 5 1
9 1 5 1
10 1 8 1
11 1 8 1
12 1 8 1
13 1 8 1
14 1 11 1
15 1 11 1
16 1 12 1
17 1 12 1
18 1 15 1
19 1 17 1
20 1 22 1
21 1 23 1
22 2 6 0
23 2 6 1
24 2 6 1
25 2 6 1
26 2 7 1
27 2 9 0
28 2 10 0
29 2 10 1
30 2 11 0
31 2 13 1
32 2 16 1
33 2 17 0
34 2 19 0
35 2 20 0
```

```
36 2 22 1
37 2 23 1
38 2 25 0
39 2 32 0
40 2 32 0
41 2 34 0
42 2 35 0
end
*to “stset” for survival analysis in Stata
stset week, failure(relapse) id(id)
*to illustrate the log-log plots
stphplot, by(group) nonegative
*to plot the plot of Schoenfeld residuals and its test against the null hypothesis
stcox group
estat phtest, plot (group)
estat phtest
*to split the data table at 10 weeks
stsplit time, at(0,10)
*to conduct the Cox regression analysis with the interaction term between the exposure status and time
stcox group##time
*to newly input the  “Gehan survival data” (because the aforementioned data are already split)
clear
input id group week relapse
1 1 1 1
2 1 1 1
3 1 2 1
4 1 2 1
5 1 3 1
6 1 4 1
7 1 4 1
8 1 5 1
9 1 5 1
10 1 8 1
11 1 8 1
12 1 8 1
13 1 8 1
14 1 11 1
15 1 11 1
```

```
16 1 12 1
17 1 12 1
18 1 15 1
19 1 17 1
20 1 22 1
21 1 23 1
22 2 6 0
23 2 6 1
24 2 6 1
25 2 6 1
26 2 7 1
27 2 9 0
28 2 10 0
29 2 10 1
30 2 11 0
31 2 13 1
32 2 16 1
33 2 17 0
34 2 19 0
35 2 20 0
36 2 22 1
37 2 23 1
38 2 25 0
39 2 32 0
40 2 32 0
41 2 34 0
42 2 35 0
end
*to "stset" for survival analysis in STATA
stset week, failure(relapse) id(id)
*to split the data table at every week
stsplit time, at(0(1)36)
*to conduct the Cox regression analysis with the interaction term between the exposure status and time
stcox group##c.time
```

***Situation 3: dealing with time-varying exposures for descriptive and predictive purposes**

***An example if the exposure of interest was a binary variable**

*to input a portion of the "BMT" dataset by Zabor, that is the first 18 of 137 patients with leukemia

```
receiving bone marrow transplant
clear
input my_id T1 delta1 TA deltaA
1 2081 0 67 1
2 1602 0 1602 0
3 1496 0 1496 0
4 1462 0 70 1
5 1433 0 1433 0
6 1377 0 1377 0
7 1330 0 1330 0
8 996 0 72 1
9 226 0 226 0
10 1199 0 1199 0
11 1111 0 1111 0
12 530 0 38 1
13 1182 0 1182 0
14 1167 0 39 1
15 418 1 418 0
16 417 1 417 0
17 276 1 276 0
18 156 1 28 1
end
*to "stset" for survival analysis in Stata
stset T1, failure(delta1) id(my_id)
*to split the follow-up time when the exposure of interest (acute graft-versus-host disease) occurred
stsplit agvhd=TA, at(0)
replace agvhd=agvhd+1
*order and rename the columns
order my_id _t0 _t agvhd delta1
keep my_id _t0 _t agvhd delta1
rename _t0 tstart
rename _t tstop
rename delta1 death
recode death .=0
```

***An example if the exposure of interest was a continuous variable**

```
*to input a hypothetical annual health survey dataset
clear
```

```
input ID BMI2010 BMI2011 BMI2012 BMI2013 BMI2014 death d_year
1 23.5 23.4 23.3 23.4 23.5 0 .
2 26.6 26.5 24.3 22.1 21.5 1 2015
3 22.1 22.3 22.5 22.2 22.0 0 .
4 20.0 18.8 17.5 . . 1 2013
5 28.5 27.8 28.0 27.7 26.6 0 .
end
*to reshape the data table to that with multiple rows per person
reshape long BMI, i(ID) j(year)
*to drop the rows if the survey year was after the patient died
drop if year>=d_year
*to replace the "death" for patients with death to have 1 only at the last row and 0 for other rows
replace death=0 if year+1!=d_year
drop d_year
*to "stset" for survival analysis in Stata
gen start=year
gen end=year+1
stset end, enter(start) origin(start) failure(death) id(ID)
*to conduct an unadjusted Cox regression analysis to estimate a weighted average of time window-specific hazard ratios of BMI at each survey year on death next year (Note: the result is not meaningfully obtained because the sample size is too small in the present hypothetical dataset)
stcox BMI
```

***Situation 5: comparing different time periods within the same individual in self-controlled case series analyses**

```
*to input the "MMR and meningitis in Oxford" dataset by Farrington et al.
clear
input indiv eventday cutp1 cutp2 exday
1 398 365 730 458
2 413 365 730 392
3 449 365 730 429
4 455 365 730 433
5 472 365 730 432
6 474 365 730 395
7 485 365 730 470
8 524 365 730 496
9 700 365 730 428
10 399 365 730 716
```

```
end
*to create cut points
gen exday14= exday+14
gen exday35= exday+35
gen age1half = 547
*to split (reshape) the follow-up time at the cut points
gen date1=cutp1
gen date2=exday14
gen date3=exday35
gen date4=age1half
reshape long date, i(indiv) j(type)
*to label the “type” to suggest what each row means
label define labelname 1"cutp1" 2"ex14" 3"ex35" 4"age1half"
label values type labelname
*to order rows according to time
sort indiv date
*to drop rows occurring after the end of study period (i.e., day 730)
drop if date>=730
*to make “start” and “end” for each row
rename date start
by indiv: gen end = start[_n+1]
*to replace the “end” of the last row with “cutp2” (i.e., the end of study period, that is day 730)
replace end=cutp2 if end==.
*to identify the interval including the “eventday”
gen nevents=0
replace nevents=1 if start<=eventday & eventday<end
*to identify the interval(s) including the risk period (i.e., between 14 and 35 days after the vaccination date)
gen exgr=0
replace exgr=1 if exday14<=start & end<=exday35
*to identify the interval(s) after the date of one and half years
gen agegr =0
replace agegr=1 if age1half<=start
*to calculate the length of each interval
gen interval = end-start
*to keep and order the necessary columns
keep indiv nevents interval agegr exgr
order indiv nevents interval agegr exgr
```

```
*to omit if interval=0 (to prevent an error in conducting a conditional Poisson regression analysis)
drop if interval==0
*to conduct a conditional Poisson regression analysis
generate loginterval = log(interval)
xi: xtpoisson nevents i.exgr i.agegr, fe i(indiv) offset(loginterval) irr
```

eMaterial 2. Commands for R (version 4.4.0)

```
# Situation 1: estimating outcome incidence rates and their ratios according to specific follow-up time periods
# to install and library required packages
if(!require(tidyverse)){install.packages("tidyverse")}
if(!require(mStats)){install.packages("mStats")}
if(!require(epitools)){install.packages("epitools")}
if(!require(flexsurv)){install.packages("flexsurv")}
if(!require(devtools)){install.packages("devtools")}


library(tidyverse)
library(survival)
library(mStats)
library(epitools)
library(flexsurv)
library(devtools)
devtools::install_github("kassambara/survminer", build_vignettes = FALSE)
library(survminer)

# to input the "Gehan survival data"
id <- seq(42)
group <- rep(c(1,2), each=21)
week <- c(1, 1, 2, 2, 3, 4, 4, 5, 5, 8, 8, 8, 8, 11, 11, 12, 12, 15, 17, 22, 23, 6, 6, 6, 6, 7, 9, 10, 10, 11, 13, 16, 17, 19, 20, 22, 23, 25, 32, 32, 34, 35)
relapse <- c(1, 1, 1, 1, 1, 1, 1, 1, 1, 1, 1, 1, 1, 1, 1, 1, 1, 1, 1, 1, 1, 0, 1, 1, 1, 1, 0, 0, 1, 0, 1, 1, 0, 0, 0, 1, 1, 0, 0, 0, 0, 0)

df <- data.frame(id=id, group=group, week=week, relapse=relapse)
head(df)

# to estimate the crude incidence rate overall
strate(df, time=week, var=relapse)

# to estimate the crude incidence rate by exposure status
strate(df, time=week, var=relapse, group)
```

```
# to illustrate Kaplan Meyer survival curves
surv_obj <- Surv(df$week, event=df$relapse)
ggsurvplot(survfit(surv_obj~df$group), data=df, risk.table=TRUE)

# to conduct a log-rank test
survdiff(surv_obj~group, data=df)

# to estimate a crude rate ratio in an unadjusted Poisson regression analysis
glm(relapse~factor(group)+offset(log(week)), data=df, family = poisson(link="log")) %>% summary()

# to estimate a crude hazard ratio in an unadjusted Cox regression analysis
model_coxph <- coxph(surv_obj~group, data=df, ties="breslow")
model_coxph %>% summary()

# to split the data table at 10 weeks
df_split10 <- survSplit(Surv(week, relapse)~group, df, cut=10,
   id="id", episode="timegroup")

# to repeat the aforementioned analyses before 10 weeks
subset0 <- df_split10 %>% dplyr::filter(tstart==0)
strate(data=subset0, time=week, var=relapse, group)
flexsurvreg(Surv(week, relapse)~factor(group), data=subset0, dist="exp")
coxph(Surv(week, relapse)~group, data=subset0, ties="breslow") %>%
   summary()

# to repeat the aforementioned analyses after 10 weeks
subset10 <- df_split10 %>% dplyr::filter(tstart==10) %>%
   mutate(obs = week-tstart)
strate(data=subset10, time=obs, var=relapse, group)
flexsurvreg(Surv(obs, relapse)~factor(group), data=subset10, dist="exp")
coxph(Surv(obs, relapse)~group, data=subset10, ties="breslow") %>%
   summary()
```

# Situation 2: assessing the proportional hazards assumption in Cox models

```
# to illustrate the log-log plots
ggsurvplot(survfit(surv_obj~df$group), data=df, fun="cloglog")
```

```
# to plot the plot of Schoenfeld residuals and its test against the null hypothesis
fit_test <- cox.zph(model_coxph, transform="identity")
fit_test
ggcoxzph(fit_test)

# to conduct the Cox regression analysis with the interaction term between the exposure status and time
coxph(Surv(tstart, week, relapse)~group*strata(timegroup), data=df_split10,
   ties="breslow") %>% summary()

# to split the data table at every week
df_split_every <- survSplit(Surv(week, relapse)~group, df,
   cut=seq(1, 36, 1), id="id", episode="timegroup")

# to conduct the Cox regression analysis with the interaction term between the exposure status and time
coxph(Surv(tstart, week, relapse)~group*timegroup, data=df_split_every,
   ties="breslow") %>% summary()
```

# Situation 3: dealing with time-varying exposures for descriptive and predictive purposes

# An example if the exposure of interest was a binary variable

```
# to input a portion of the "BMT" dataset by Zabor, that is the first 18 of 137 patients with leukemia receiving bone marrow transplant
my_id <- seq(18)
T1 <- c(2081, 1602, 1496, 1462, 1433, 1377, 1330, 996, 226, 1199, 1111,
   530, 1182, 1167, 418, 417, 276, 156)
delta1 <- c(0, 0, 0, 0, 0, 0, 0, 0, 0, 0, 0, 0, 0, 0, 1, 1, 1, 1)
TA <- c(67, 1602, 1496, 70, 1433, 1377, 1330, 72, 226, 1199, 1111, 38, 1182,
   39, 418, 417, 276, 28)
deltaA <- c(1, 0, 0, 1, 0, 0, 0, 1, 0, 0, 0, 1, 0, 1, 0, 0, 0, 1)

df_BMT <- data.frame(my_id=my_id, T1=T1, delta1=delta1, TA=TA,
   deltaA=deltaA)
head(df_BMT)

# to split the follow-up time when the exposure of interest (acute graft-versus-host disease) occurred
df_id <- df_BMT %>% filter(my_id==1)
df_split_id <- survSplit(Surv(T1, delta1)~my_id+deltaA, data=df_id,
   cut=df_id$TA, episode="timegroup", end="tend")
```

```
df_split_BMT <- df_split_id

for (i in 2:18) {
  df_id <- df_BMT %>% filter(my_id==i)
  df_split_id <- survSplit(Surv(T1, delta1)~my_id+deltaA, df_id,
      cut=df_id$TA, episode="timegroup", end="tend")
  df_split_BMT <- bind_rows(df_split_BMT, df_split_id)
}

# to create the time-dependent variable of the exposure of interest(agvhd)
df_split_BMT <- df_split_BMT %>%
    mutate(agvhd = case_when((timegroup==2 & deltaA==1)~1, TRUE~0))

# order and rename the columns
df_split_BMT <- df_split_BMT %>%
    rename(death = delta1) %>%
    select(my_id, tstart, tend, agvhd, death)
head(df_split_BMT)
```

# An example if the exposure of interest was a continuous variable

```
# to input a hypothetical annual health survey dataset
ID <- seq(5)
BMI2010 <- c(23.5, 26.6, 22.1, 20, 28.5)
BMI2011 <- c(23.4, 26.5, 22.3, 18.8, 27.8)
BMI2012 <- c(23.3, 24.3, 22.5, 17.5, 28)
BMI2013 <- c(23.4, 22.1, 22.2, NA, 27.7)
BMI2014 <- c(23.5, 21.5, 22, NA, 26.6)
death <- c(0, 1, 0, 1, 0)
d_year <- c(NA, 2015, NA, 2013, NA)

df3 <- data.frame(ID=ID, BMI2010=BMI2010, BMI2011=BMI2011, BMI2012=BMI2012,
    BMI2013=BMI2013, BMI2014=BMI2014, death=death, d_year=d_year)
head(df3)

# to reshape the data table to that with multiple rows per person
#to replace the "death" for patients with death to have 1 only at the last row and 0 for other rows
#to drop the rows if the survey year was after the patient died
df3_long <- df3 %>%
```

```
  pivot_longer(cols=starts_with("BMI"), names_to="year", values_to="BMI",
    names_prefix="BMI") %>%
  mutate(year = as.numeric(year)) %>%
  mutate(death = case_when(d_year<=year+1~1, TRUE~0)) %>%
  select(-d_year) %>%
  filter(!is.na(BMI))
head(df3_long)

df3_long <- df3_long %>% mutate(start = year, end = year+1)

# to conduct an unadjusted Cox regression analysis to estimate a weighted average of time window-specific hazard ratios of BMI at each survey year on death next year
# (Note: the result is not meaningfully obtained because the sample size is too small in the present hypothetical dataset)
coxph(Surv(start, end, death==1)~BMI+cluster(ID), robust=TRUE,
  data=df3_long, method="breslow")
```

# Situation 5: comparing different time periods within the same individual in self-controlled case series analyses

```
# to input the "MMR and meningitis in Oxford" dataset by Farrington et al.
indiv <- seq(10)
eventday <- c(398, 413, 449, 455, 472, 474, 485, 524, 700, 399)
cutp1 <- rep(365, 10)
cutp2 <- rep(730, 10)
exday <- c(458, 392, 429, 433, 432, 395, 470, 496, 428, 716)

df_MMR <- data.frame(indiv=indiv, eventday=eventday, cutp1=cutp1,
  cutp2=cutp2, exday=exday)
head(df_MMR)

# to create cut points
df_MMR <- df_MMR %>% mutate(exday14 = exday+14,
                            exday35 = exday+35,
                            age1half = 547)

# to split (reshape) the follow-up time at the cut points
df_MMR <- df_MMR %>% mutate(date1 = cutp1,
```

```
                                        date2 = exday14,
                                        date3 = exday35,
                                        date4 = age1half)

df_MMR_long <- df_MMR %>%
  pivot_longer(cols=starts_with("date"), names_to="type", values_to="date",
    names_prefix="date")
head(df_MMR_long)

# to drop rows occurring after the end of study period (i.e., day 730)
df_MMR_long <- df_MMR_long %>% filter(date < 730)

# to make "start" and "end" for each row
# to replace the "end" of the last row with "cutp2" (i.e., the end of study period, that is day 730)
df_MMR_long <- df_MMR_long %>%
  arrange(indiv, date) %>%
  group_by(indiv) %>%
  mutate(start = date, end =lead(start)) %>%
  mutate(end = case_when(is.na(end)~cutp2, TRUE~end))

# to identify the interval including the "eventday"
#to identify the interval(s) including the risk period (i.e., between 14 and 35 days after the vaccination
date)
#to calculate the length of each interval
df_MMR_long <- df_MMR_long %>%
  mutate(nevents = case_when((start<=eventday & eventday<end)~1, TRUE~0),
          exgr = case_when((exday14<=start & end<=exday35)~1, TRUE~0),
          agegr = case_when(age1half<=start~1, TRUE~0),
          interval = end-start)

# to keep and order the necessary columns
df_MMR_ana <- df_MMR_long %>% select(indiv, nevents, interval, agegr, exgr)

# to omit if interval=0 (to prevent an error in conducting a conditional Poisson regression analysis)
df_MMR_ana <- df_MMR_ana %>% filter(interval > 0)

# to conduct a conditional Poisson regression analysis
clogit(nevents~factor(exgr)+factor(agegr)+strata(indiv)+offset(log(interval)),
```

```
data=df_MMR_ana) %>%
  summary()
```

eMaterial 3. Commands for SAS (version 9.4)

```
/************************************************************
Situation 1: estimating outcome incidence rates and their ratios according to specific follow-up time periods;
************************************************************/

/* To input the "Gehan survival data" */
data gehan;
    input id group week relapse;
        logweek = log(week);
    datalines;
1 1 1 1
2 1 1 1
3 1 2 1
4 1 2 1
5 1 3 1
6 1 4 1
7 1 4 1
8 1 5 1
9 1 5 1
10 1 8 1
11 1 8 1
12 1 8 1
13 1 8 1
14 1 11 1
15 1 11 1
16 1 12 1
17 1 12 1
18 1 15 1
19 1 17 1
20 1 22 1
21 1 23 1
22 2 6 0
23 2 6 1
24 2 6 1
25 2 6 1
26 2 7 1
```

```
27 2 9 0
28 2 10 0
29 2 10 1
30 2 11 0
31 2 13 1
32 2 16 1
33 2 17 0
34 2 19 0
35 2 20 0
36 2 22 1
37 2 23 1
38 2 25 0
39 2 32 0
40 2 32 0
41 2 34 0
42 2 35 0
;
run;

/* To illustrate Kaplan-Meier survival curves */
/* To conduct a log-rank test */
proc lifetest data = gehan plots = (s) graphics;
    time week * relapse(0);
    strata group;
run;

/* To estimate the crude incidence rate by exposure status */
* Person-time method;
proc means data = gehan noprint;
        var week relapse;
        class group;
        output out = rate sum = py ev;
run;

data rate;
        set rate;
        rate = ev/py;
        rate_95LL = exp(log(rate) - 1.96 * sqrt(1/ev));
```

```
        rate_95UL = exp(log(rate) + 1.96 * sqrt(1/ev));
run;

proc print data = rate;
        var group ev py rate:;
run;

/* To replicate incidence rate via Poisson regression */
proc sort data = gehan; by group; run;

proc genmod data = gehan;
    model relapse = /dist = Poisson offset = logweek;
        by group;
        estimate "log Rate" int 1/ exp;
run;

/* To estimate rate ratio in an unadjusted Poisson regression analysis */
proc genmod data = gehan;
    class group/param = ref ref = first;
    model relapse = group / dist = Poisson offset = logweek;
        estimate "log Rate Ratio" group 1/ exp;
run;

/* To estimate a crude hazard ratio in an unadjusted Cox regression analysis */
proc phreg data = gehan;
    class group/param = ref ref = first;
    model week * relapse(0) = group /rl;
run;


/* To split the data table at 10 weeks */
proc sort data = gehan; by id; run;

data gehan_split;
    set gehan;
    if week <= 10 then do;
                time = 0;
                end = week;
```

```
                    output;
            end;
        else do time = 0, 10;
                    if time = 0 then end = 10;
                    else end = week;
                    output;
            end;
run;

data gehan_split;
            set gehan_split;
                    relapse = (end = week) * (relapse = 1);
                    week = end - time;
                    logweek = log(week);
run;

/* To repeat the aforementioned analyses before and after 10 weeks */
proc sort data = gehan_split; by time; run;

proc genmod data = gehan_split;
            by time;
        class group/ param = ref ref = first;
        model relapse = group / dist = poisson offset = logweek;
            estimate "log Rate in group = 0" int 1 group 0/ exp;
            estimate "log Rate in group = 1" int 1 group 1/ exp;
            estimate "log Rate Ratio" int 0 group 1/ exp;
            ods select Estimates;
run;

proc phreg data = gehan_split;
            by time;
        class group/ param = ref ref = first;
        model week * relapse(0) = group /rl;
run;


/*************************************************************
```

Situation 2: assessing the proportional hazards assumption in Cox models;

```
***********************************************************/

/* To set up the data for survival analysis in SAS (equivalent to stset) */
proc sort data = gehan; by id; run;

/* To illustrate the log-log plots */
proc lifetest data = gehan plots = (lls) graphics;
      time week * relapse(0);
      strata group;
run;

/* To plot the Schoenfeld residuals and test against the null hypothesis */
proc phreg data = gehan zph(transform= identity);
      class group;
      model week * relapse(0) = group;
            assess ph;
      output out = residuals ressch = schoenfeld;
run;

proc sgplot data = residuals;
      scatter x = week y = schoenfeld / group = group;
      reg x = week y = schoenfeld / group = group;
run;

proc reg data = residuals;
            model   schoenfeld = week;
run;

/* To conduct the Cox regression analysis with the interaction term between the exposure status and
time */
proc phreg data = gehan_split;
      class group time/ param = ref ref = first;
      model week * relapse(0) = group group*time/ rl;
            strata time;
            estimate "log HR before time 10" group 1/ exp cl;
            estimate "log HR after time 10" group 1 group*time 1/ exp cl;
            estimate "log Ratio of HRs" group*time 1/ exp cl;
run;
```

```
*To conduct the Cox regression analysis with the interaction term between the exposure status and time;
proc phreg data = gehan;
	class group/ param = ref ref = first;
	model week * relapse(0) = group group*time_at_event/ rl;
		time_at_event = week;
		estimate "log Ratio in HRs per week" group*time_at_event 1/ exp cl;
run;
```

```
/************************************************************
```

Situation 3: dealing with time-varying exposures for descriptive and predictive purposes;

```
************************************************************/
```

***An example if the exposure of interest was a binary variable;**

```
*to input a portion of the "BMT" dataset by Zabor, that is the first 18 of 137 patients with leukemia receiving bone marrow transplant;
data bmt;
	input my_id T1 delta1 TA deltaA;
	datalines;
1 2081 0 67 1
2 1602 0 1602 0
3 1496 0 1496 0
4 1462 0 70 1
5 1433 0 1433 0
6 1377 0 1377 0
7 1330 0 1330 0
8 996 0 72 1
9 226 0 226 0
10 1199 0 1199 0
11 1111 0 1111 0
12 530 0 38 1
13 1182 0 1182 0
14 1167 0 39 1
15 418 1 418 0
16 417 1 417 0
17 276 1 276 0
18 156 1 28 1
```

```
;
run;

*Split the follow-up time when the exposure of interest occurred;
data bmt_split;
    set bmt;
        if deltaA = 1 then do;
        tstart = 0;
        tstop = TA;
        agvhd = 0;
        death = 0;
        output;

        tstart = TA;
        tstop = T1;
        agvhd = 1;
        death = delta1;
        output;
    end;
    else do;
        tstart = 0;
        tstop = T1;
        agvhd = 0;
        death = delta1;
        output;
    end;
        keep my_id tstart agvhd tstop death;
run;
```

***An example if the exposure of interest was a continuous variable;**

```
*to input a hypothetical annual health survey dataset;

data health;
    input ID BMI2010 BMI2011 BMI2012 BMI2013 BMI2014 death d_year;
    datalines;
1 23.5 23.4 23.3 23.4 23.5 0 .
2 26.6 26.5 24.3 22.1 21.5 1 2015
```

```
3 22.1 22.3 22.5 22.2 22.0 0 .
4 20.0 18.8 17.5 . . 1 2013
5 28.5 27.8 28.0 27.7 26.6 0 .
;
run;

*Reshape the data table to that with multiple rows per person;
proc transpose data = health out = health_long(rename = (col1 = BMI));
    by ID death d_year;
    var BMI2010-BMI2014;
run;

data health_long;
        format ID year BMI death;
    set health_long;
        year = input(substr(_NAME_, 4.), 4.);

        *Drop the rows if the survey year was after the patient died;
        if 0 < d_year <= year then delete;

        *Replace the "death" for patients with death to have 1 only at the last row and 0 for other rows:;
    if death = 1 and year = d_year - 1 then death = 1;
    else death = 0;

        drop d_year _NAME_;
run;

*Set up for survival analysis;
data health_long;
    set health_long;
    start = year;
    end = year + 1;
run;

proc sort data = health_long; by ID start; run;

proc phreg data = health_long;
    class ID;
```

```
    model (start, end) * death(0) = BMI / ties = efron;
    id ID;
run;


/*********************************************************
Situation 5: comparing different time periods within the same individual in self-controlled case series
analyses;
*********************************************************/

/* Input the "MMR and meningitis in Oxford" dataset by Farrington et al. */
data mmr;
    input indiv eventday cutp1 cutp2 exday;
    datalines;
1 398 365 730 458
2 413 365 730 392
3 449 365 730 429
4 455 365 730 433
5 472 365 730 432
6 474 365 730 395
7 485 365 730 470
8 524 365 730 496
9 700 365 730 428
10 399 365 730 716
;
run;

/* Create cut points */
data mmr;
    set mmr;
    exday14 = exday + 14;
    exday35 = exday + 35;
    age1half = 547;
run;

/* Split (reshape) the follow-up time at the cut points */
proc transpose data = mmr out = mmr_long;
        by indiv;
```

```
        var cutp1 exday14 exday35 age1half;
run;

data mmr_long;
        merge mmr mmr_long(rename = (_NAME_ = type col1 = date));
        by indiv;
        label type =;
run;

/* Order rows according to time */
proc sort data = mmr_long;
        by indiv date;
run;

/* Create start and end for each row */
data mmr_long;
        merge mmr_long(rename = (date = start)) mmr_long(firstobs = 2 rename = (indiv = indiv_
date = end) keep = indiv date);
        if indiv ^= indiv_ then end = cutp2;
        drop indiv_;
/* Drop rows occurring after the end of study period (i.e., day 730) */
        if cutp2 <= start then delete;
run;

data mmr_long;
        set mmr_long;

/* Identify the interval including the eventday */
        if start <= eventday < end then nevents = 1;
        else nevents = 0;

/* Calculate the length of each interval */
        interval = end - start;
        if interval > 0 then log_interval = log(interval);

/* Identify the interval(s) after the date of one and half years */
        if age1half <= start then agegr = 1;
        else agegr = 0;
```

```
/* Identify the interval(s) including the risk period (i.e., between 14 and 35 days after the vaccination
date) */
            if exday14 <= start and end <= exday35 then exgr = 1;
            else exgr = 0;

            keep indiv nevents interval log_interval agegr exgr;
run;

/* Conduct a conditional Poisson regression analysis (All model fit returns the same results) */
proc genmod data = mmr_long;
            where interval > 0;
      class indiv;
      model nevents = exgr agegr indiv/ dist = poisson offset = log_interval;
            estimate "log conditional maximum-likelihood rate ratio"exgr 1/ exp;
run;

proc logistic data = mmr_long desc;
            where interval > 0;
            model nevents = exgr agegr/ offset = log_interval;
            strata indiv;
run;

proc phreg data = mmr_long;
            where interval > 0;
            model indiv * nevents(0) = exgr agegr/ offset = log_interval rl; * The time variable can be any
value that is constant within an individual case;
            strata indiv;
run;
```
